# Supplementary material for: Unique Interactions of the Small Translocases of the Mitochondrial Inner Membrane (Tims) in Trypanosoma brucei
Source: Int J Mol Sci. 2024 Jan 24;25(3):1415. doi: 10.3390/ijms25031415 (PMC10855554; doi:10.3390/ijms25031415)
Supplement: Supplementary file 1 [file ijms-25-01415-s001.zip › ijms-2707982-supplementary.pdf]

Supplementary materials

Unique Interactions of the Small Translocases of the Mitochondrial Inner Membrane (Tims) in *Trypanosoma brucei*

Linda S. Quiñones[1], Fidel Soto Gonzales[1⊢], Chauncey Darden[2], Muhammad Khan[1ˀ], Anuj Tripathi[1ᵛ], Joseph T. Smith Jr.[3], Jamaine Davis[2], Smita Misra[4], Minu Chaudhuri[1]*

[1]Department of Microbiology, Immunology, and Physiology, School of Medicine, Meharry Medical College, Nashville, Tennessee, USA

[2] Department of Biochemistry, Cancer Biology, Neuroscience, and Pharmacology, School of Medicine, Meharry Medical College, Nashville, Tennessee, USA

[3]Department of Microbiology and Immunology, Jacobs School of Medicine and Biomedical sciences, University at Buffalo, Buffalo, NY

[4]Department of Biomedical Science, School of Graduate Studies, Meharry Medical College, Nashville, Tennessee, USA

Current address: ⊢NIH/NIDDK, Bethesda, MD; ᵛBundelkhand University, Jhansi, India.

*Address correspondence to Minu Chaudhuri, mchaudhuri@mmc.edu

Department of Microbiology, Immunology, and Physiology, Meharry Medical College, Nashville, TN, USA

Phone: (615) 327–5726

Email: mchaudhuri@mmc.edu

Table S1. Primers used in this study

Primer Name	DNA Sequence (5'-3')
TbTim9-HA Forward	GATCAAGCTTATGCGCCTGGCTG
TbTim9-HA Reverse	GATCTCTAGACAACTTCAACATTTG
TbTim10-HA Forward	GATCAAGCTTATGCAGCCACCTC
TbTim10-HA Reverse	GATCTCTAGATTCGTTCTCCGAC
TbTim8/13-HA Forward	GATCAAGCTTATGAATCAGTCTAGTTC
TbTim8/13-HA Reverse	GATCTCTAGACCTTTCCTTTGCTTG
TbTim12-HA Forward	GATCAAGCTTATGGGTCAGGACCAATCC
TbTim12-HA Reverse	GATCTCTAGAACGACTAAGTTCCTTTC
TbTim13-HA Forward	GATCAAGCTTATGCAACCCCCAACCCCAC
TbTim13-HA Reverse	GATCTCTAGAGACCCCTCCCCCCTG
TbTim11 RNAi Forward	GATCAAGCTTATGCAGAGCCAAATGATGC
TbTim11 RNAi Reverse	GATCGGATCCCTACTCCTCCATACCGAGGGATC
TbTim12 RNAi Forward	GATCAAGCTTATGGGTCAGGACCAATCC
TbTim12 RNAi Reverse	GATCGGATCCTTAACGACTAAGTTCCTTTC
TbTim13 RNAi Forward	GATCAAGCTTATGCAACCCCCAACC
TbTim13 RNAi Reverse	GATCGGATCCCTAGACCCCTCCCCCCTG
TbTim11-qRT Forward	TTTTGCCGGTCAAACAGAGC
TbTim11-qRT Reverse	CCAGCCACAAGTGATTGAAGTG
TbTim12-qRT Forward	ATTACCCTTCGGCGTCAAGG
TbTim12-qRT Reverse	GCTTTCACCCAGTCTCCGAA
TbTim13-qRT Forward	GGCGAACTGGCAGTGATAGT
TbTim13-qRT Reverse	CGCTACTCTTGCAACCCTCA
Tubulin-qRT Forward	TTCCGCACCCTGAAACTGA
Tubulin-qRT Reverse	TGACGCCGGACACAACAG

Restriction enzyme sites are underlined

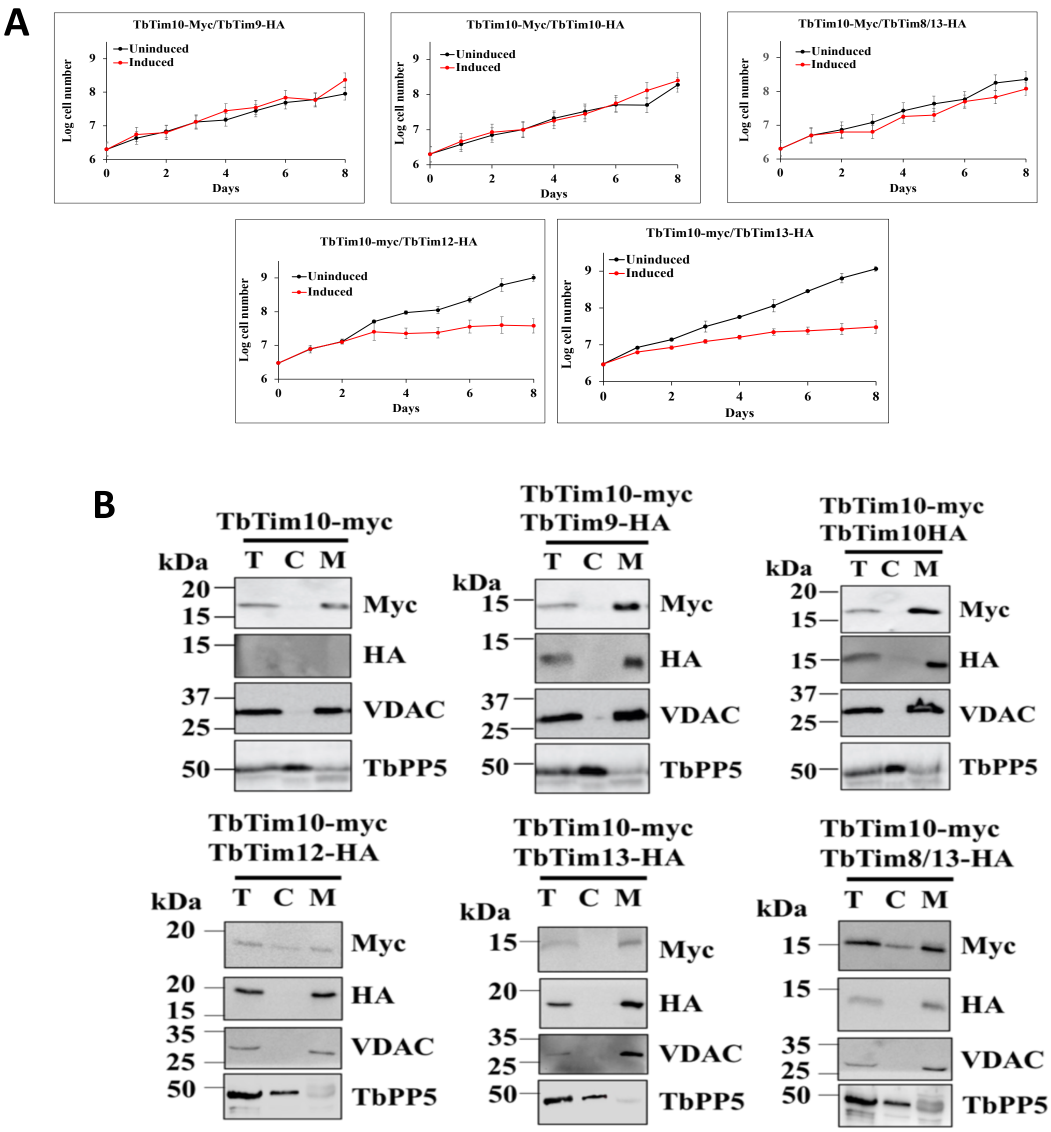

Figure S1. Effect of overexpression of small TbTims on cell growth and localization. *T. brucei* cell line that expresses TbTim10-2X-Myc in a tetracycline inducible fashion was transfected individually with TbTim9-3X-HA, TbTim10-3X-HA, TbTim8/13-3X-HA, TbTim12-3X-HA, TbTim13-3X-HA constitutively expression constructs to generate double-tagged small TbTims cell lines. (A) Cell growth analysis of *T. brucei* expressed TbTim10-Myc+TbTim9-HA, TbTim10-Myc+TbTim10-HA, TbTim10-Myc+TbTim8/13-HA, TbTim10-Myc+TbTim12-HA, and TbTim10-Myc+TbTim13-HA parasite in the absence (Uninduced) and presence (Induced) of doxycycline (1.0 μg/ml) for 8 days. The log of the cumulative cell numbers was plotted versus days post-induction. (B) Cellular localization of the tagged small TbTims. Each cell line was induced for 2 days with doxycycline and subcellular fractions were collected. Proteins from the total cell lysates (T), cytosol (C), and mitochondrial fractions (M) were subjected to SDS-PAGE and were probed with anti-Myc and anti-HA antibody. VDAC and TbPP5 were used as the mitochondrial and cytosolic marker proteins, respectively. Samples were run in more than one gel, therefore results for each cell type are presented in individual panel.

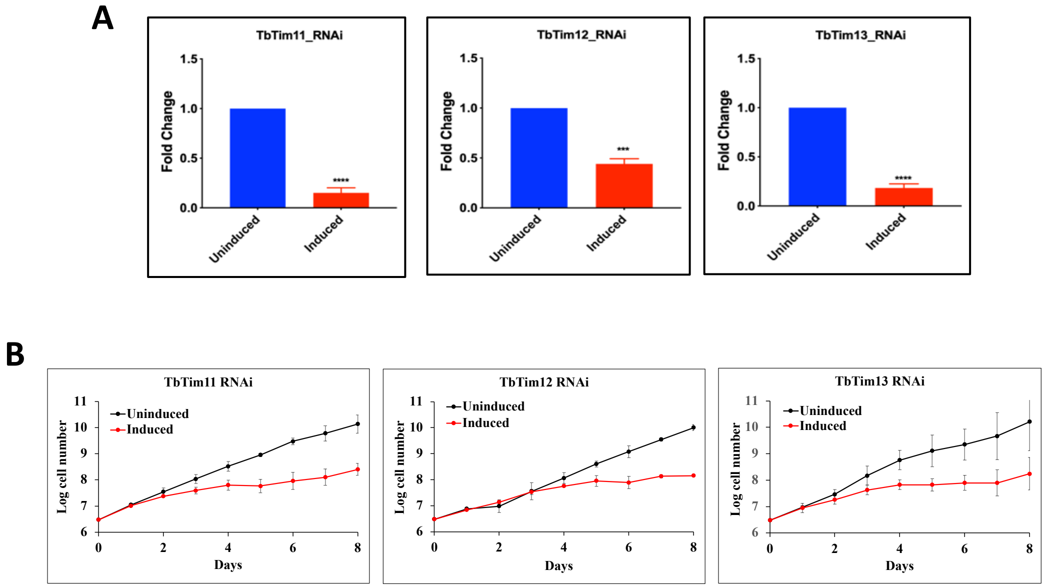

Figure S2. Effect of small TbTim RNAi on the levels of specific transcripts and cell growth in *T. brucei*. A) qRT-PCR analysis of TbTim11, TbTim12, TbTim13 mRNA levels in TbTim11 RNAi, TbTim12 RNAi, TbTim13 RNAi *T. brucei* cells were grown in the absence (Uninduced) and presence of doxycycline (1.0 μg/ml) for 48 h. The mRNA levels of the target transcript in the uninduced cells were considered as 100%. All transcript levels were normalized with the levels of the tubulin mRNA in each sample. Values were calculated from three independent replicates and are plotted with error bars for standard deviations. Pvalues are 0.0001 (****) and 0.0004 (***). Raw data are available in the supplemental file. B) Cell growth analysis of TbTim11, TbTim12, and TbTim13 RNAi parasite in the absence (Uninduced) and presence (Induced) of doxycycline (1.0 μg/ml) for 8 days. The log of the cumulative cell numbers was plotted versus days post-induction. Standard errors were calculated from three independent experiment.

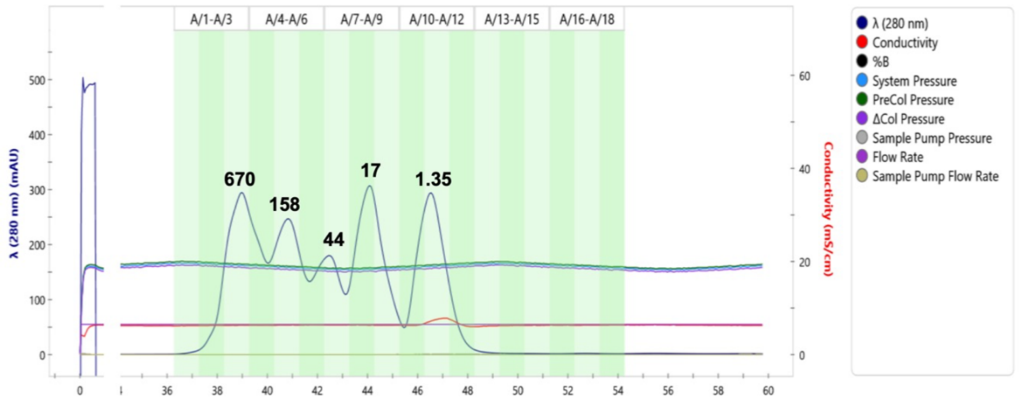

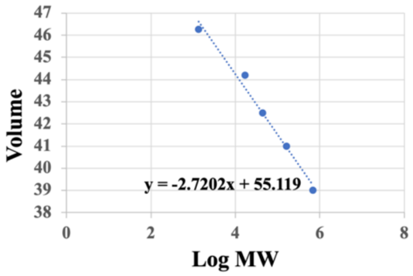

Fraction (Vol)	kDa
Frac2 (38 v)	1964
Frac4 (40 v)	361
frac 6 (42 v)	66.5
frac 7 (43 v)	28.5

Figure S3. Standard protein run on SEC650. Standard molecular marker protein mixture (BioRad) (200 µl) was loaded on a ENrich^{TM} SEC 650 10 X 300 column (BioRad) and run on an NGC system (BioRad). Proteins were eluted with 1X Native buffer (20 mM Tris, pH 7.0, 50 mM NaCl, 1 mM EDTA, 10% glycerol, 1 mM PMSF, and 0.1% digitonin). Peaks for known molecular weight standards; Thyroglobulin, 670 kDa; γ-globulin, 158 kDa; Ovalbumin, 44 kDa; Myoglobin 17 kDa, and vitamin B12, 1.35 kDa are marked. Proteins were identified by color and by expected molecular sizes on a denaturing gel. A standard curve was generated by plotting elution volumes of the peaks versus log of molecular weight of the standard proteins. Unknown molecular weight of the mitochondrial protein complexes eluted at different volumes were calculated and shown in the table.
